# Redo Surgery after Failed Open VBG: Laparoscopic Minigastric Bypass versus Laparoscopic Roux en Y Gastric Bypass—Which Is Better?

**DOI:** 10.1155/2016/8737519

**Published:** 2016-05-29

**Authors:** Tamer M. S. Salama, Karim Sabry

**Affiliations:** Department of General Surgery, Faculty of Medicine, Ain Shams University, Abbassia Square, Cairo 3435544, Egypt

## Abstract

*Background*. Long-term studies have reported that the rate of conversion surgeries after open VBG ranged from 49.7 to 56%. This study is aiming to compare between LMGB and LRYGB as conversion surgeries after failed open VBG with respect to indications and operative and postoperative outcomes.* Methods*. Sixty patients (48 females and 12 males) presenting with failed VBG, with an average BMI of 39.7 kg/m^2^ ranging between 26.5 kg/m^2^ and 53 kg/m^2^, and a mean age of 38.7 ranging between 24 and 51 years were enrolled in this study. Operative and postoperative data was recorded up to one year after the operation.* Results*. MGB is a simple procedure that is associated with short operative time and low rate of complications. However, MGB may not be applicable in all cases with failed VBG and therefore RYGB may be needed in such cases.* Conclusion*. LMGB is a safe and feasible revisional bariatric surgery after failed VBG and can achieve early good weight loss results similar to that of LRYGP. However, the decision to convert to lap RYGB or MGB should be taken intraoperatively depending mainly on the actual intraoperative pouch length.

## 1. Introduction

Vertical banded gastroplasty (VBG) was one of the most commonly performed bariatric surgeries in the last decade [1]. However, in the following years the operation did not achieve optimum results as it was associated with long-term weight gain and some mechanical complications. Later, long-term studies have reported that the rate of conversion surgeries after open VBG ranged from 49.7 to 56% [2, 3].

Over the past years, RYGB is the most commonly performed conversion surgery after failed open VBG as it achieves good long-term results in weight loss. However, it is associated with a high rate of complications and long-term metabolic side effects [4].

As a primary bariatric surgery, minigastric bypass which was first described by Rutledge was found to achieve excellent results with short operative duration and low rates of postoperative complications [5, 6].

This study is aiming to compare between LMGB and LRYGB as conversion surgeries after failed open VBG with respect to indications and operative and postoperative outcomes.

## 2. Patients and Methods

Sixty patients (48 females and 12 males) presenting with failed VBG, an average BMI of 39.7 kg/m^2^ ranging between 26.5 kg/m^2^ and 53 kg/m^2^, and a mean age of 38.7 ranging between 24 and 51 years were enrolled in this prospective randomized study.

Patients were admitted at the Bariatric Unit, Department of General Surgery, El Demerdash Hospital, at Ain Shams University in Cairo, Egypt, from December 2013 to December 2015. Approval from the Ethical Committee of the Faculty of Medicine at Ain Shams University was obtained to conduct this study.

All patients enrolled in this study were suffering from failed VBG, that is, weight loss of less than 50% of the excess body weight in 2 years and/or having VBG related complications such as stomal stenosis with persistence vomiting, resistant stomal ulcers, intractable bleeding, severe reflux esophagitis, pouch dilation or staple line disruption, GG fistula with weight regain, or poor control of obesity-associated comorbidities.

Patients with severe debilitating nutritional deficiency, large incisional hernias, history of personality disorder, drug or alcohol addiction, or advanced malignancy were excluded from our study. Additionally, patients who were contraindicated for laparoscopy or general anesthesia (e.g., having major medical comorbidity such as cardiac patients) or refused the laparoscopic procedure were also excluded.

Before the operation, assessment of patients' general conditions, mental statuses, and obesity-associated comorbidities such as diabetes, hypertension, or cardiovascular diseases was performed, in addition to nutritional assessment for vitamin B12, calcium, magnesium, iron and protein, fat, and carbohydrate body composition.

Full preoperative work-up including blood chemistries, ultrasonography, barium meal, and upper endoscopy was performed for all patients.

All patients wrote an informed consent before the operation after they were provided with a full and clear explanation of benefits, risks, and long-term consequences of the conversion to bypass surgery. During the week prior to surgery, patients were instructed to eat a high protein diet and perform regular exercises, while during the day before operation, they were allowed to only take clear fluids. The procedure was performed by laparoscopy.

Intraoperatively, patients were intubated in a supine position and pneumoperitoneum was established through a 10 mm umbilical Visiport. One 5 mm trocar was placed under xiphoid process for the insertion of the liver retractor, 12 and 15 mm trocars were placed on the right and left middle clavicular lines few millimeters above the umbilicus, respectively, for the surgeon instruments, and another 5 mm trocar was placed on the left anterior axillary line for assistance. Oral Ryle was inserted to deflate the stomach to facilitate the dissection. As a first step, we tried to separate the stomach wall from the left lobe of the liver and overlying omentum in an attempt to identify the site of the mesh. Then, bougie with a size of 36 was inserted into the stomach. If it passed easily and freely without gastric outlet obstruction, the mesh was not removed and the operation was continued as MGB in which the first transverse staple line was placed at the level of the incisura and vertical stapling on the previous VBG staple line was then placed (in this case, the pouch was usually not dilated). If gastric outlet obstruction was found and did not allow the bougie to pass, the mesh was attempted to be removed without injuring the gastric wall. In case we succeeded and the bougie was passed easily, MGB was performed as described above. If we failed, the 1st transverse reload was to be taken just above the mesh and proceeded vertically to the angle of hiss. If the vertical length of the gastric pouch was long (enough to take 3 reloads each of size 60 mm), the operation was continued as MGB where after bypassing 180 cm of intestine from the ligament of Treitz in an anticolic fashion, loop gastroenterostomy was performed.

In cases where the vertical length of the pouch was short (less than 3 reloads each of a size 60), the operation was continued as RYGB where the biliopancreatic limb was 70 cm and the alimentary limb was 150 cm using a linear stapler to create end to side gastrojejunostomy and side to side jejunojejunostomy. Both enterotomies closed by V-lock and the mesenteric defects closed by nonabsorbable prolene 2/zero.

Hemostasis was assessed and staple line was checked using methylene blue. Then, a tube drain with a size of 22 was inserted routinely and was removed 2 to 3 days after the operation.

Postoperative standard clinical protocol was used for all patients. All patients were on “nil by mouth” for 48 hours followed by low-caloric clear liquids for 1 week and low-caloric semisolid food for 2–4 weeks postoperatively. Full diet was subsequently introduced. Patients were discharged from the hospital in the 3rd day after Gastrografin study was performed. Patients were followed up once every week for one month and then once every month for one year to monitor their postoperative outcome as regards general health condition, BMI, and complication.

## 3. Results

Sixty patients (48 females and 12 males) complaining from failed VBG with a mean age of 38.7 years (ranging from 24 to 51 years) and an average BMI of 39.7 kg/m^2^ (ranging from 26.5 kg/m^2^ to 53 kg/m^2^) were enrolled in this study (Table 1).

In the current study, 70% of our patients were complaining from failing to achieve satisfactory weight loss or having weight regain after open VBG, while the remaining 30% were complaining from other VBG complications such as persistent vomiting, reflux esophagitis, or attacks of bleeding.

MGB with long gastric pouch was successfully performed in 39 cases and mesh was removed in 15 cases. The mean duration of intervention was 145 min (ranging from 125 to 235 min) and the mean length of hospital stay was 4.7 days (ranging from 4 to 18 days). The mean BMI decreased to 30.1 kg/m^2^ (ranging from 24.8 kg/m^2^ to 41.5 kg/m^2^) after 1 year of the operation. One case had leakage after 2 days of the operation and upon performing reexploration, an iatrogenic injury in the ascending limb of omega loop in the MGB was found. This perforation was closed by a primary suture (Table 2).

RYGB with short gastric pouch was performed in 21 cases with a mean duration of operation of 185 min (ranging from 130 to 312 min) and a mean length of hospital stay of 6.2 days (ranging from 5 to 7 days). The mean BMI decreased to 29.8 kg/m^2^ (ranging from 24.3 to 40.3 kg/m^2^) after 1 year of the operation. One case had anastomotic stenosis in the gastrojejunostomy in the 8th month after the operation which was improved after balloon dilatation. Another case had intestinal obstruction and upon reexploration, hernia through the mesenteric defect was found. The herniated intestine was viable and reduction with closure of the defect was performed. Finally, no mortalities occurred in this study.

## 4. Discussion

In the past decade, vertical band gastroplasty was amongst the preferred bariatric surgeries for weight loss without being associated with metabolic side effects. However, the procedure did not provide satisfactory long-term weight loss results as more than 20% of patients regained their weight after surgery. Weight regain after failed VBG was attributed to staple line disruption, pouch dilation, and the switch in patients eating habits to become “sweet eaters” [7]. According to a study performed by Van Gemert et al. [2], up to 56% of patients who underwent VBG would require revisional surgery over a period of 12 years.

RYBG was the revisional surgery of choice after failed VBG is RYBG since it can achieve good results in weight loss and permits corrections of comorbidities [8]. However, revisional LRYGB is a technically difficult procedure and is associated with higher morbidities and mortalities [9].

LRYBG is considered a technically difficult procedure because of the high anastomosis near the esophagogastric junction which necessitates the complete release of the upper stomach which can be a highly difficult and risky step. Moreover, the high anastomosis near the esophagogastric junction can be under tension and may cause fistula formation [10].

Developments made in laparoscopic revisional bariatric surgeries led to the arising of LMGB as a safer substitute to LRYBG as it does not require the complete release of the upper stomach as the anastomosis is performed inferiorly so it is enough to create retrogastric tunnel for the stapler under direct vision guided by the bougie for stapler. Moreover, LMGB is superior in the fact that it is associated with single anastomosis with better blood supply for gastric tube decreasing the risk of leakage [11].

This study is subsequently addressing if LMGB is a legitimate revisional procedure for all cases with failed VBG. We found that long pouch was successfully created after the spontaneous passage of the bougie through the stoma which occurred in 18 cases or after the removal of the mesh which occurred in 15 cases or due to the presence of dilation in the upper gastric pouch which is commonly associated with stomal stenosis as found in 6 cases. This enabled us to convert VBG into MGB in 65% of our patients, while in 35% of the patients long pouch could not be created and the vertical length of the pouch was less than 3 reloaded of size 60 mm and therefore VBG was converted into RYGB to avoid reflux esophagitis. This indicates that not all cases with failed VBG can be converted into MGB and sometimes it is much better for the patients to convert into RYGB. This decision should be taken intraoperatively.

The mean operative time and mean postoperative hospital stay in the cases converted to MGB were 145 min and 4.7 days, respectively, which were significantly shorter in comparison to cases converted into RYGB where the mean operative time and mean postoperative hospital stay were 185 min and 6.2 days, respectively. These results reflect the simplicity of MGB in comparison to RYGB.

One year after the operation, there was no significant difference between the postoperative mean BMI of cases converted into MGB (30.1 kg/m^2^) and that of cases converted into RYGB (29.8 kg/m^2^) indicating that both procedures have similar weight loss efficiencies.

There was a significant decrease in the rate of complications after MGB in comparison to RYGB which was 2.5% and 9.5%, respectively. After MGB, there was only one case out of the 39 cases that had leakage which was a traumatic injury due to hard grasping of the intestinal loop and not due to leakage from the gastrojejunostomy anastomosis, while after RYGB one case had internal hernia and one case had stomal stenosis. In a study performed by Gonzalez et al. [12], they stated that anastomotic strictures and leaks are relatively high after revisional LRYGB. Additionally, another study performed by Gagné et al. [13] stated that strictures are common complication after revisional LRYGB and it occurs because of proximal gastric pouch mucosal thickening or distal pouch ischemia due to chronic inflammation from vertical staple line.

Therefore we can state that MGB is a simple procedure that is associated with short operative and low rate of complications. However, MGB may not be applicable in all cases with failed VBG and therefore RYGB may be needed in such cases.

## 5. Conclusion

LMGB is a safe and feasible revisional bariatric surgery after failed VBG and can achieve early good weight loss results similar to that of LRYGP. However, the decision to convert to lap RYGB or MGB should be taken intraoperatively depending mainly on the actual intraoperative pouch length.

## Figures and Tables

**Table 1:** The demographic and preoperative data of patients.

Gender *N* (%)	Male	12	20%
	Female	48	80%

Age	Range	24	51
	Mean ± SD	38.733	10.062

BMI kg/m^2^ before redo surgery	Range	26.5	53
	Mean ± SD	39.792	8.212

Cause of failure of VBG *N* (%)	Regain weight	42	70%
	Other VBG complications	18	30%

Mesh (%)	Removed	15 in case	25%

**Table 2:** The operative and postoperative data of patients.

		MGB		RYGB		*P* value	Sig.
Number of cases		39	65%	21	35%		

Duration of intervention	Range	125–235		130–312		0.011	S
	Mean ± SD	145.410 ± 29.184		185.162 ± 57.777			

Length of hospital stay	Range	4–18		5–7		0.004	S
	Mean ± SD	4.769 ± 2.241		6.286 ± 0.717			

BMI kg/m^2^ after the operation	Range	24.8–41.5		24.3–40.3		0.654	NS
	Mean ± SD	30.154 ± 5.362		29.886 ± 5.689			

Complications		1 case	2.56%	2 cases	9.52%	0.576	NS

## References

[1] Mason E. E., Tang S., Renquist K. E. (1997). A decade of change in obesity surgery. *Obesity Surgery*.

[2] Van Gemert W. G., van Wersch M. M., Greve J. W. M., Soeters P. B. (1998). Revisional surgery after failed vertical banded gastroplasty: restoration of vertical banded gastroplasty or conversion to gastric bypass. *Obesity Surgery*.

[3] Miller K., Pump A., Hell E. (2007). Vertical banded gastroplasty versus adjustable gastric banding: prospective long-term follow-up study. *Surgery for Obesity and Related Diseases*.

[4] Wylezol M., Pardela M. (2005). Results of revisional operations following vertical banded gastroplasty performed due to morbid obesity—comparison between restoration of vertical banded gastroplasty and conversion to gastric bypass up to three years. *Journal of Physiology and Pharmacology*.

[5] Rutledge R. (2001). The mini-gastric bypass: experience with the first 1,274 cases. *Obesity Surgery*.

[6] Lee W.-J., Yu P.-J., Wang W., Chen T.-C., Wei P.-L., Huang M.-T. (2005). Laparoscopic Roux-en-Y versus mini-gastric bypass for the treatment of morbid obesity: a prospective randomized controlled clinical trial. *Annals of Surgery*.

[7] Wang W., Huang M.-T., Wei P.-L., Chiu C.-C., Lee W.-J. (2004). Laparoscopic mini-gastric bypass for failed vertical banded gastroplasty. *Obesity Surgery*.

[8] Khaitan L., Van Sickle K., Gonzalez R. (2005). Laparoscopic revision of bariatric procedures: is it feasible?. *The American Surgeon*.

[9] Ortega J., Sala C., Flor B. (2004). Vertical banded gastroplasty converted to Roux-en-Y gastric bypass: little impact on nutritional status after 5-year follow-up. *Obesity Surgery*.

[10] Suter M., Giusti V., Héraief E., Calmes J.-M. (2004). Band erosion after laparoscopic gastric banding: occurrence and results after conversion to Roux-en-Y gastric bypass. *Obesity Surgery*.

[11] Noun R., Zeidan S., Riachi E., Abboud B., Chalhoub V., Yazigi A. (2007). Mini-gastric bypass for revision of failed primary restrictive procedures: a valuable option. *Obesity Surgery*.

[12] Gonzalez R., Gallagher S. F., Haines K., Murr M. M. (2005). Operative technique for converting a failed vertical banded gastroplasty to Roux-en-Y gastric bypass. *Journal of the American College of Surgeons*.

[13] Gagné D. J., Dovec E., Urbandt J. E. (2011). Laparoscopic revision of vertical banded gastroplasty to Roux-en-Y gastric bypass: outcomes of 105 patients. *Surgery for Obesity and Related Diseases*.

